# Supplementary material for: Identifying cell states in single-cell RNA-seq data at statistically maximal resolution
Source: PLoS Comput Biol. 2024 Jul 12;20(7):e1012224. doi: 10.1371/journal.pcbi.1012224 (PMC11364423; doi:10.1371/journal.pcbi.1012224)
Supplement: S1 Text — (PDF) [file pcbi.1012224.s001.pdf]

Identifying cell states in single-cell RNA-seq data at statistically maximal resolution

Pascal Grobecker[1], Thomas Sakoparnig[1], Erik van Nimwegen[1,*]

1 Biozentrum, University of Basel and Swiss Institute of Bioinformatics, Basel, Switzerland

* erik.vanimwegen@unibas.ch

Supporting Information

Contents

A Detailed Derivations **1**
A1 Likelihood of partitions . . . 1
A2 Posterior of transcription quotients . . . 4
A3 Cellstate similarities and hierarchical clustering . . . 5
A4 Differentially expressed genes between pairs of cellstate clusters . . . 5
A5 Marker genes for distinguishing between pairs of cellstate clusters . . . 7

B Computational Methods **9**
B1 MCMC Algorithm for maximizing the likelihood . . . 9
B2 Simulated Datasets . . . 10
B3 Further Discussion of the results on simulated data . . . 10

C Benchmarking of other clustering tools **11**

D Substructure within cell types found by Cellstates does not correspond to batch effects **11**

E Supplementary Figures **13**

A Detailed Derivations

A1 Likelihood of partitions

We consider an scRNA-seq dataset D characterized by a matrix of UMI counts n_{gc} corresponding to the number of UMIs for gene g in cell c. We denote by ρ partitions of the cells into non-overlapping subsets and want to determine a likelihood function $P(D|\rho)$ under the assumption that, for each subset $s \in \rho$ of the partition, all cells $c \in s$ have the same gene expression state. To explain how this likelihood function is calculated, we first discuss how the mRNA counts m_g of a cell, and observed UMI counts n_g in an scRNA-seq experiment depend on the gene expression processes in the recent history of the cell, as previously introduced in [1].

Given the inherent thermodynamic fluctuations affecting the molecules inside the cell, and the Brownian motion that they are subject to, even a comprehensive description of the current 'state' of the cell in terms of the number of molecules of each type in each cellular compartment only determines the *rates* with which different molecular reactions occur. For the mRNA levels of a given gene g, the relevant rates are the transcription rate λ_g and the mRNA decay-rate μ_g. It is well established that for a gene g with constant transcription rate λ_g and constant mRNA decay-rate μ_g, the number of mRNA molecules in

the cell m_g follows a Poisson distribution with mean $\langle m_g \rangle = a_g = \lambda_g/\mu_g$, e.g. [2]. More generally, when μ_g and λ_g are arbitrary time-dependent functions $\mu_g(t), \lambda_g(t)$, with $\lambda_g(t)$ denoting the transcription rate a time t in the past of the cell, and $\mu_g(t)$ the decay rate of mRNAs for gene g a time t in the past, the probability distribution for the current number of mRNAs m_g in the cell is still a Poisson distribution with mean [1]:

$$a_g = \langle m_g \rangle = \int_0^\infty dt \lambda_g(t) \exp\left[-\int_0^t \mu_g(s) ds\right], \tag{1}$$

which we call the 'transcription activity' of gene g. Note that time is measured backwards from the present ($t = 0$) to the distant past ($t = \infty$) in the history of the cell. Thus, a single parameter a_g for each gene g is sufficient to fully characterize the distribution of mRNA numbers in a cell at any given time point. The remaining uncertainty about the actual numbers is due to random thermodynamic fluctuations in events such as RNA polymerase binding or mRNA degradation. To conclude, given the expression state of the cell as defined by the vector of transcription activities $\vec{a}$, the probability of a count vector of cellular mRNAs across all genes $\vec{m}$ is therefore a product of Poisson distributions:

$$P(\vec{m}|\vec{a}) = \prod_g \frac{(a_g)^{m_g}}{m_g!} e^{-a_g}. \tag{2}$$

We will assume that the *measured* UMI counts n_g correspond to a random sample of the cell's total mRNA pool m_g with some unknown capture rate p per mRNA. As will be discussed below, our model remains valid if the capture rate p varies between cells or has gene-dependent biases. Given these assumptions, the likelihood for the observed UMI count vector $\vec{n}$ is still a Poisson distribution, albeit with a different mean:

$$P(\vec{n}|\vec{a}, p) = \prod_g \frac{(pa_g)^{n_g}}{n_g!} e^{-pa_g}. \tag{3}$$

Following [1], we now define the transcription quotients $\alpha_g = a_g/A$, with $A = \sum_g a_g$ the total transcription activity of the cell. Note that α_g corresponds to the expected fraction of transcripts from gene g among all transcripts in the cell. For a cell with a total UMI count $N = \sum_g n_g$, we have

$$\langle n_g \rangle = \alpha_g pA = \alpha_g N. \tag{4}$$

Conditioned on the total count N, the distribution of all measured counts $\vec{n}$ is a multinomial in the transcription quotients:

$$P(\vec{n}|\vec{\alpha}, N) = N! \prod_g \frac{1}{n_g!} (\alpha_g)^{n_g} \propto \prod_g (\alpha_g)^{n_g}. \tag{5}$$

This is the form of the likelihood of the UMI counts of a single cell as a function of the transcription quotient vector $\vec{\alpha}$.

In our model, we use the transcription quotient vector $\vec{\alpha}$ to represent the 'expression state' of a cell and the key ingredient of our model is that, given a partition ρ, all cells within each subset s of the partition have the same transcription quotient vector $\vec{\alpha}$. The likelihood for the counts D_s of a subset of cells s that have equal transcription quotients $\vec{\alpha}$, is given by

$$P(D_s|\vec{\alpha}) = \prod_{c \in s} P(\vec{n_c}|\vec{\alpha}) \propto \prod_g (\alpha_g)^{n_{gs}} \tag{6}$$

where $\vec{n_s} = \sum_{c \in s} \vec{n_c}$ is the vector of total UMI counts among all cells in the subset s.

To calculate the likelihood $P(D|\rho)$ of a partition, we will marginalize over the unknown transcription quotient vector $\vec{\alpha}$ for each of the subsets s in ρ and to do this we have to define a *prior* distribution over possible transcription quotient vectors $\vec{\alpha}$. We will use a Dirichlet prior, which corresponds to a maximal

ignorance prior in the sense that it is the unique prior that is invariant under arbitrary rescaling of the transcription quotients $\alpha_g \to \lambda_g \alpha_g$. Moreover, it is the conjugate prior to the multinomial distribution, allowing us to analytically marginalize over the $\vec{\alpha}$. In particular, for each subset s we characterize our prior information regarding its transcription quotient vector $\vec{\alpha}$ by the *same* Dirichlet prior:

$$P(\vec{\alpha}|\vec{\theta}) = \frac{\Gamma(\Theta)}{\prod_g \Gamma(\theta_g)} \prod_g (\alpha_g)^{\theta_g - 1}, \tag{7}$$

where the θ_g are the parameters of the Dirichlet prior and $\Theta = \sum_g \theta_g$. We can now marginalize over $\vec{\alpha}$ and obtain

$$P(D_s|\vec{\theta}) = \int_{\sum_g \alpha_g = 1} P(D_s, \vec{\alpha}|\vec{\theta})\, d\vec{\alpha} \tag{8}$$

$$= \int_{\sum_g \alpha_g = 1} P(D_s|\vec{\alpha}) P(\vec{\alpha}|\vec{\theta})\, d\vec{\alpha} \tag{9}$$

$$= \frac{\Gamma(\Theta)}{\Gamma(N_s + \Theta)} \prod_g \frac{\Gamma(n_{gs} + \theta_g)}{\Gamma(\theta_g)} \tag{10}$$

with $N_s = \sum_g n_{gs}$ is the total number of UMI summed over all cells in subset s. The likelihood of a partition ρ is now obtained by simply taking the product of this expression over all subsets $s \in \rho$:

$$P(D|\rho, \vec{\theta}) = \prod_{s \in \rho} P(D_s|\vec{\theta}) \tag{11}$$

$$= \prod_{s \in \rho} \left[\frac{\Gamma(\Theta)}{\Gamma(N_s + \Theta)} \prod_g \frac{\Gamma(n_{gs} + \theta_g)}{\Gamma(\theta_g)} \right]. \tag{12}$$

Note that this expression is very similar to likelihood functions derived previously for clustering DNA sequences [3]. Essentially the only change is that the 4-letter DNA alphabet is here replaced by the 'alphabet' of G genes.

Using a uniform prior on the partitions ρ and the parameters $\vec{\theta}$ of the Dirichlet priors, the posterior distribution $P(\rho, \vec{\theta}|D)$ is now simply proportional to the likelihood $P(D|\rho, \vec{\theta})$. Ideally we would further marginalize over the parameters $\vec{\theta}$ to obtain a posterior distribution over partitions only, but this is analytically intractable. We thus aim to find the combination $(\rho, \vec{\theta})$ that jointly maximizes both the posterior $P(\rho, \vec{\theta}|D)$ and likelihood $P(D|\rho, \vec{\theta})$, i.e. optimizing over both the partition ρ and the parameters θ_g for each gene individually. Without loss of generality, we can rewrite the parameters of the prior θ_g as the product of an overall scale vector Θ and a normalized vector $\vec{\phi}$ with $\sum_g \phi_g = 1$. Second, we note that for the trivial partition in which all cells are put into a single cluster, the optimal ϕ_g are given by

$$\phi_g = \frac{\sum_c n_{gc}}{\sum_{c,g} n_{gc}}, \tag{13}$$

i.e. the prior parameter ϕ_g simply equals the fraction of UMIs for gene g in the entire dataset. In order to keep the optimization numerically tractable, we will approximate the optimization of the prior's parameters by fixing $\vec{\phi}$ to this vector, setting $\vec{\theta} = \Theta\vec{\phi}$, and only optimize the scale factor $\Theta \in \mathbb{R}^+$, while leaving the ϕ_g fixed for a given dataset. Setting the prior in this way ensures that, for each subset s, the expected direction of the transcription quotient vector $\vec{\alpha}$ matches the overall UMI counts in the entire dataset, while optimizing Θ allows to tuning of the expected amount of variability around this 'average' vector of transcription quotients.

With this chosen form of the prior, we finally get an expression for the likelihood of the whole dataset D that only depends on the scale factor Θ and the partition of cells into subsets ρ:

$$P(D|\rho,\Theta) = \prod_{s\in\rho} P(D_s|\Theta) \tag{14}$$

$$= \prod_{s\in\rho}\left[\frac{\Gamma(\Theta)}{\Gamma(N_s+\Theta)}\prod_g \frac{\Gamma(n_{gs}+\Theta\phi_g)}{\Gamma(\Theta\phi_g)}\right]. \tag{15}$$

Finally, we return to discuss our simplifying assumption that the mRNA capture rate p is constant across genes and cells and show that this assumption can be significantly relaxed without affecting the results. In particular, we will assume that the probability p_{gc} of capturing (and successfully amplifying and sequencing) an mRNA for gene g in cell c can be written as

$$p_{gc} = p_c q_g, \tag{16}$$

where p_c is a cell-specific overall capture rate and q_g describes gene-dependent biases that may be specific to the particular experiment, but are assumed constant across the cells in the experiment. With this capture efficiency, the expected UMI count for gene g in cell c is $\langle n_{gc}\rangle = p_c q_g a_g$. Thus, the expected fraction of counts from gene g becomes

$$\frac{\langle n_{gc}\rangle}{N_c} = \frac{a_g p_c q_g}{\sum_g a_g p_c q_g} = \frac{a_g q_g}{\sum_g a_g q_g} = \tilde{\alpha_g}, \tag{17}$$

where the last equality defines $\tilde{\alpha}_g$. From here, we can proceed the derivation exactly as before with $\tilde{\alpha_g}$ replacing α_g. As we marginalize out this variable in Equation (10), the final result is invariant. Note that, given that we separately marginalize over the $\vec{\alpha_g}$ of each subset, the result is even invariant when different subsets s have different gene-bias vectors $\vec{q}$, as long as all cells within a subset have the same bias. This suggests that our likelihood over partitions is not only insensitive to fluctuations in overall capture efficiency across cells, but will also be quite robust to fluctuations in gene-dependent capture efficiency as long as cells with equal expression states have equal biases.

A2 Posterior of transcription quotients

Although for calculating likelihoods over partitions ρ, we marginalize over the GES $\vec{\alpha_s}$ for each subset s, we can of course also obtain posterior distributions over these GES for each cluster. Given a subset of cells s, the posterior distribution for its GES $\vec{\alpha_s}$ can be obtained from equations (6), (7) and (10) to find:

$$P(\alpha_s|D_s,\Theta) = \frac{P(\alpha_s|\Theta)P(D_s|\alpha_s)}{P(D_s|\Theta)} = \frac{\Gamma(\Theta+N_s)}{\prod_g \Gamma(n_{gs}+\Theta\phi_g)}\prod_g (\alpha_g)^{\Theta\phi_g+n_{gs}-1}. \tag{18}$$

From this expression, we can derive expressions for mode, mean and variance of $\vec{\alpha_s}$:

$$\text{Mode}[\alpha_{gs}] = \frac{\max(\Theta\phi_g+n_{gs}-1,0)}{\sum_g \max(\Theta\phi_g+n_{gs}-1,0)} \tag{19}$$

$$\langle\alpha_{gs}\rangle = \frac{\Theta\phi_g+n_{gs}}{\Theta+N_s} \tag{20}$$

$$\text{Var}[\alpha_{gs}] = \frac{(\Theta\phi_g+n_{gs})(\Theta(1-\phi_g)+N_s-n_{gs})}{(\Theta+N_s)^2(\Theta+N_s+1)} \tag{21}$$

Often we are interested in the log-transcription quotients $\delta_{gs} = \log(\alpha_{gs})$ rather than the transcription quotients themselves. For these we find:

$$\text{Mode}[\delta_{gs}] = \log(\Theta\phi_g + n_{gs}) - \log(\Theta + N_s) \tag{22}$$

$$\langle \delta_{gs} \rangle = \psi(\Theta\phi_g + n_{gs}) - \psi(\Theta + N_s) \tag{23}$$

$$\text{Var}[\delta_{gs}] = \psi_1(\Theta\phi_g + n_{gs}) + \psi_1(\Theta + N_s), \tag{24}$$

where ψ is the digamma function (the derivative of the logarithm of the Gamma function) and ψ_1 is the first derivative of the digamma function.

Table A. Summary of most important variables used in this section.

a_g	Transcription activity for gene g
m_g	Number of mRNAs of gene g in a cell
n_g	Number of UMIs measured for gene g in a cell
α_g	Transcription quotient for gene g
θ_g	Parameters of the Dirichlet prior, $\theta_g = \Theta\phi_g$
Θ	Scale of Dirichlet prior parameters
ϕ_g	Unit vector of Dirichlet prior parameter
D	Dataset, full table of UMI counts n_g
ρ	Partition of cells into a set of subsets s

A3 Cellstate similarities and hierarchical clustering

We define the similarity between two subsets of cells S_a and S_b as the ratio of the likelihoods of the partitions with both subsets merged and each subset separate:

$$\begin{aligned}\sigma_{ab} =& \frac{P(D|\rho(S_a + S_b), \Theta)}{P(D|\rho(S_a, S_b), \Theta)} \\ =& \frac{P(\vec{n_a} + \vec{n_b}|\Theta\vec{\phi})}{P(\vec{n_a}|\Theta\vec{\phi})P(\vec{n_b}|\Theta\vec{\phi})}\end{aligned} \tag{25}$$

Note that this similarity metric does not behave like a usual distance metric in that similar clusters will have a large σ and dissimilar clusters a small σ close to 0. To build a hierarchical tree of the cellstates, we start by setting the leaf clusters of the tree to the cellstates of the optimal partition and then calculate all pairwise similarities σ_{ab} between all pairs of leaf clusters. We then iteratively merge the pair of clusters with the highest similarity, and recalculate the pairwise similarities with the newly formed cluster until all clusters have merged into a single cluster. For plotting, we save the resulting tree in the Newick format with distances set to positive log-similarities $d_{ab} = \max[-\log(\sigma_{ab}), 0]$.

A4 Differentially expressed genes between pairs of cellstate clusters

To describe the differences between the GES of different clusters, and to help give biological interpretation, it is useful to quantify which genes are most significantly differentially expressed between the clusters. In our framework, we can quite naturally define the extent of differential expression of genes by decomposing Equation (25) into contributions of individual genes, i.e. $\sigma_{ab} = \prod_g \sigma_{ab,g}$. A low value of $\sigma_{ab,g}$ for a gene g indicates that the UMI counts are much more different between the two clusters a and b than would be

expected from noise. A high value $\sigma_{ab,g}$, in contrast, indicates that counts are within the expected noise levels.

To obtain such a decomposition, we start by decomposing the cluster likelihood of Equation (10) into contributions from individual genes $P(\vec{n}|N,\Theta) = \prod_g P(n_g|N,\Theta)$. In the multinomial model, the likelihood is conditioned on the total number of captured mRNA in the cell N, so that, formally, the counts n_{gc} are correlated for all pairs of genes. However, since this correlation is generally quite weak, we can make the assumption that the expression noise is independent between genes. Thus,

$$P(\vec{n},\vec{\alpha}|\Theta) \approx \prod_g P(n_g,\alpha_g|\Theta) \tag{26}$$

$$= \prod_g P(n_g|\alpha_g)P(\alpha_g|\Theta). \tag{27}$$

We can find $P(\vec{n}|\alpha_g)$ by marginalizing over the other variables:

$$P(\vec{n}|\alpha_g) \propto \int_{\sum_{g'\neq g}\alpha_{g'}=1-\alpha_g} P(\vec{n}|\vec{\alpha})P(\{\alpha_{g'\neq g}\}|\alpha_g,\vec{\theta})\,\{d\alpha_{g'\neq g}\} \tag{28}$$

$$\propto (\alpha_g)^{n_g}(1-\alpha_g)^{N-n_g} \tag{29}$$

where $N = \sum_g n_g$. We can further marginalize over $\{n_{g'\neq g}\}$, requiring that N is constant, and normalize to obtain the binomial distribution:

$$P(n_g|\alpha_g,N) = \binom{N}{n}(\alpha_g)^{n_g}(1-\alpha_g)^{N-n_g}. \tag{30}$$

Next, we find $P(\alpha_g|\Theta)$:

$$P(\alpha_g|\Theta) = \int_{\sum_{g'\neq g}\alpha_{g'}=1-\alpha_g} P(\vec{\alpha}|\Theta)\,d\vec{\alpha} \tag{31}$$

$$= \frac{1}{B(\Theta\phi_g,\Theta(1-\phi_g))}(\alpha_g)^{\Theta\phi_g-1}(1-\alpha_g)^{\Theta(1-\phi_g)-1}, \tag{32}$$

where $B(\alpha,\beta) = \Gamma(\alpha)\Gamma(\beta)/\Gamma(\alpha+\beta)$.

Finally, we have all ingredients of Equation (27) above. Unlike in Equation (10) where we perform the integral subject to the constraint $\sum_g \alpha_g = 1$, we integrate over all α_g separately. With the high dimensionality of $\vec{\alpha}$ and assuming the likelihood function has a sharp peak, the error will be small.

$$P(\vec{n}|\theta) \propto \prod_g \int_0^1 P(n_g|\alpha_g)P(\alpha_g|\vec{\theta})\,d\alpha_g \tag{33}$$

$$= \prod_g \int_0^1 \frac{1}{B(\theta_g,\Theta-\theta_g)}(\alpha_g)^{n_g+\theta_g-1}(1-\alpha_g)^{N-n_g+\Theta-\theta_g-1}\,d\alpha_g \tag{34}$$

$$= \prod_g \frac{B(n_g+\theta_g,N-n_g+\Theta-\theta_g)}{B(\theta_g,\Theta-\theta_g)} \tag{35}$$

This likelihood function clearly has a separate contribution $P(n_g|N,\Theta)$ for each gene:

$$P(n_g|N,\Theta) = \frac{B(n_g + \Theta\phi_g, N - n_g + \Theta(1-\phi_g))}{B(\Theta\phi_g, \Theta(1-\phi_g))} \tag{36}$$

$$= \frac{\Gamma(\Theta)}{\Gamma(\Theta(1-\phi_g))\Gamma(\Theta\phi_g)} \frac{\Gamma(n_g + \Theta\phi_g)\Gamma(N - n_g + \Theta(1-\phi_g))}{\Gamma(N+\Theta)} \tag{37}$$

Comparing to Equation (10), we see that this is equivalent to taking the ratio of the cluster likelihood with gene g and without g.

Finally, we can use this expression for $P(n_g|N,\Theta)$ in Equation (25) and define a gene-specific score for differential expression

$$\sigma_{ab,g} = \frac{P(n_{a,g} + n_{b,g}|N_a + N_b, \Theta)}{P(n_{a,g}|N_a,\Theta)P(n_{b,g}|N_b,\Theta)}, \tag{38}$$

where the subscripts a, b refer to two subsets of cells. Genes with $\sigma_{ab,g} < 1$ have counts that poorly fit a model with a single transcription quotient for both clusters, compared to a model where the two clusters have distinct transcription quotients. In contrast, genes with a score $\sigma_{ab,g} > 1$ favour a model with a single transcription quotient and are not differentially expressed between the clusters.

A5 Marker genes for distinguishing between pairs of cellstate clusters

In the previous section we derived a score $\sigma_{ab,g}$ that quantifies how significant the difference is in expression of gene g between the set of cellstates a and the set of cellstates b. Importantly, this significance measure depends on the *average* expression of gene g in the sets a and b and is independent of how the expression of gene g varies across cells in the sets a and b. Consequently, a gene g can be highly significant even if the distributions of its expression level across the cells of sets a and b are highly overlapping.

Thus, besides a method for finding genes whose average expression differs most significantly between the cells of a and b, it is also useful to have a method for identifying genes whose expression can reliably distinguish cells from set a from cells of set b. That is, we want to identify genes whose distributions of expression levels across sets a and b exhibit *least overlap*.

Given two sets of objects a and b, and a real-valued quantity x that is given for each object, we want to define a measure that quantifies how well the variable x can classify objects from set a from those of set b. A reasonable measure for this classification ability is the area under the ROC curve which is obtained by varying a cut-off q and plotting the fraction of objects in a that have $x < q$ on the vertical axis (often called true positive rate), and the fraction of objects in b that have $x < q$ (false positive rate) on the horizontal axis. Notably, this area under the ROC is equal to the probability that if we pick one random object o_a from set a and one random object o_b from set b, that the x value of o_a is less than the x value of o_b. A variable x is a good classifier when this probability is either near 1 (i.e. the x values of the a objects are systematically below the x values of the b objects) or near 0 (when the x values of the a objects are systematically larger than those of the objects in b).

We will use this measure to quantify to what extent the expression of a given gene g separates cells in set a from cells in set b. The complication is that we do not know the precise expression levels of each of the cells. Instead, for a cell from cellstate c, we only have a posterior distribution $P(\alpha_g|c)$ for the transcription quotient of gene g. Thus, if we define $|c|$ as the number of cells in cell state c, and $P(\alpha_g|c)$ as the posterior distribution of the transcription quotient of gene g given the UMI counts of the cells in cell-state c, then the probability we want to calculate is the following:

$$P_g(a < b) = \sum_{c_1 \in a, c_2 \in b} \frac{|c_1||c_2|}{|a||b|} \int_0^1 d\alpha_g P(\alpha_g|c_2) \int_0^{\alpha_g} d\beta_g P(\beta_g|c_1), \tag{39}$$

with $|a|$ and $|b|$ the total number of cells in the sets of cellstates a and b, respectively. Thus, for each pair of cell-states (c_1, c_2) from sets a and b, we calculate the probability that the transcription quotient β_g in cell-state c_1 is less than the transcription quotient α_g in cell-state c_2. Then we take a weighted average over these pair scores, weighted with the number of cells in each cell state. This then gives the probability that if we sample a random cell from one of the cell states in a and one random cell from one of the cell states in b, that the transcription quotient α_g of gene g is lower in the first cell than in the second cell. Whenever this probability $P_g(a < b)$ is either close to zero or close to 1, then the gene g is a good marker gene for distinguishing the sets a and b.

For a given gene, let n_g be its UMI count in the cellstate c, N the total UMI count of cellstate c, θ_g the parameter of the global Dirichlet prior for gene g, and $\Theta = \sum_g \theta_g$. The posterior probability for the transcription quotient α_g in cellstate c is then given by the beta-distribution

$$P(\alpha_g|c, \theta) = \frac{\Gamma(N + \Theta)}{\Gamma(n_g + \theta_g)\Gamma(N + \Theta - n_g - \theta_g)} \alpha_g^{n_g+\theta_g-1}(1 - \alpha_g)^{N+\Theta-n_g-\theta_g-1}. \tag{40}$$

For a given pair of cell states (c_1, c_2), the integrals that we have to calculate are of the form

$$P_g(c_1 < c_2) = \int_0^1 d\alpha_{g2} P(\alpha_{g2}|c_2) \int_0^{\alpha_{g2}} d\alpha_{g1} P(\alpha_{g1}|c_1). \tag{41}$$

Although, as far as we can tell, these integrals are not analytically tractable, we can make progress by noting that, generally, the transcription quotient of a given gene g is much less than 1 in both cellstates , i.e. $\alpha_{g1} \ll 1$ and $\alpha_{g2} \ll 1$. Under this assumption, the above Beta-distributions are well approximated by Gamma-distributions:

$$P(\alpha_g|c) \propto (\alpha_g)^{n_{gc}+\theta_g-1} e^{-(N+\Theta)\alpha_g}. \tag{42}$$

If we make this approximation then the integrals can be performed analytically and the final result is a cumulative distribution of a beta-distribution:

$$P_g(c_1 < c_2) = I_{(n_{g1}+\theta_g, n_{g2}+\theta_g)} \left(\frac{N_1 + \Theta}{N_1 + N_2 + 2\Theta} \right), \tag{43}$$

where n_{g1} and n_{g2} are the UMI counts of gene g in cellstates c_1 and c_2, and N_1 and N_2 are the total UMI counts in cellstates c_1 and c_2.

Equation (43) is just the cumulative beta-distribution with parameters the UMI counts for the gene in the two cell states, i.e. $n_{g1} + \theta_g$ and $n_{g2} + \theta_g$, evaluated at a point that is the fraction of the total UMI counts that are in the first cell state, i.e. $(N_1 + \Theta)/(N_1 + N_2 + 2\Theta)$.

To calculate a final marker score S_g for a gene to distinguish between the set of cell states a and the set of cell states b we calculate the probability $P_g(a < b)$ as:

$$P_g(a < b) = \sum_{c_1 \in a, c_2 \in b} \frac{|c_1||c_2|}{|a||b|} I_{n_{gc_1}+\theta_g, n_{gc_2}+\theta_g} \left(\frac{N_{c_1} + \Theta}{N_{c_1} + N_{c_2} + 2\Theta} \right). \tag{44}$$

The function $I_{\alpha,\beta}(x)$ is the cumulative probability of the beta-distribution with parameters α and β evaluated at x.

The gene g is a good marker when $P_g(a < b)$ is either close to zero or close to one. So to get a final target score S_g we use the absolute value of the log-ratio between $P_g(a < b)$ and $1 - P_g(a < b)$

$$S_g = \left| \log \left[\frac{P_g(a < b)}{1 - P_g(a1 < b)} \right] \right|. \tag{45}$$

We have implemented this marker score in a C program that is distributed with Cellstates and have provided example notebooks of its use on the associated GitHub page.

B Computational Methods

B1 MCMC Algorithm for maximizing the likelihood

Our aim is to identify the combination of the prior's scale factor Θ and partition ρ that jointly maximize the likelihood $P(D|\rho, \Theta)$ given in Equation (14). Notably, since the likelihood function is a very complex function of both the partition ρ and the prior's scale factor Θ, this optimization requires a fairly expensive search. The general structure of our optimization algorithm is to first start with an initial guess for Θ and to then iteratively

1. Search for the partition ρ_* that maximizes the likelihood with the current value of Θ,
2. Given this optimal partition ρ_*, find the value of Θ that maximizes the likelihood $P(D|\rho_*, \Theta)$,

until Θ and ρ_* no longer change.

First, with respect to the optimization of Θ, we have found empirically that both the optimal partition ρ_* and the likelihood $P(D|\rho, \Theta)$ depend only weakly on Θ, so that the optimal partition ρ_* does not depend sensitively on Θ, and it suffices to only roughly estimate the optimal value of Θ. Since ρ has to be re-optimized for every change in Θ, we thus limit ourselves to only consider values of $\Theta = 2^q, q \in \mathbb{N}$. That is, we only estimate the optimal Θ to within a factor 2. Furthermore, for the initial value of Θ we take $q = \lfloor \log_2(\langle N_{UMI} \rangle) + 0.5 \rfloor$ where $\langle N_{UMI} \rangle$ is the average number of total UMI counts per cell. That is, our initial guess for Θ corresponds to the average total UMI count per cell. This means that strength of the influence of the prior is about equal to the influence of the data from a single-cell.

During the development of Cellstates, we have experimented with a number of different schemes for searching the optimal partition ρ. This included greedy algorithms that iteratively fuse pairs of cellstates so as to maximize the increase in the likelihood $P(D|\rho, \Theta)$ and Gibbs sampling schemes in which at each time step a random cell is chosen and then moved into a cluster with probability proportional to the likelihood of the resulting partition. However, we found that the best trade-off between running time and optimality of the final partition is obtained using a simple Markov chain Monte Carlo (MCMC) algorithm that we developed previously for sampling partitions of short DNA sequences [3].

In particular, given a value of Θ we optimize the partition by starting from the partition in which each cell forms its own cluster. Starting from this partition, we then sample the space of partitions using a Metropolis-Hastings scheme. To explain this sampling scheme, we conceptualize partitions as distributions of the C cells over C 'boxes' such that all cells in the same box form a cluster and different boxes correspond to different clusters. We thus initially assign each of the C cells to one of C boxes and the space of partitions is searched by moving cells between the C boxes. Specifically we iterate the following steps:

1. A cell is chosen uniformly at random and taken out of its current box.
2. One of the $C - 1$ other boxes is chosen uniformly at random and we consider the partition ρ' that is created by moving the cell into this box.
3. We calculate the likelihood ratio of the new to old partitions: $P_{\text{move}} = P(D|\rho', \Theta)/P(D|\rho, \Theta)$.
4. If the new partition ρ' has N_{clus} clusters and ρ had $N_{\text{clus}} + 1$ clusters, we set $P_{\text{bias}} = (C - N_{\text{clus}})$, otherwise $P_{\text{bias}} = 1$.
5. If $P_{\text{move}} * P_{\text{bias}} \geq 1$, the move is accepted. Otherwise, we draw a random number p in the range $p \in [0, 1)$ and accept the move if $p < P_{\text{move}} * P_{\text{bias}}$.

Note that the additional factor P_{bias} (which appears only when the move reduces the number of clusters) is needed to ensure detailed-balance of the chain as explained in detail in the supplementary material of [3].

These steps are iterated until the likelihood has stopped increasing for a sufficient number of steps. In the current implementation of the algorithm, the stopping criterion is controlled by two parameters: a 'step number' S and 'try number' T. Each round a total number of $S \times T$ moves are attempted. This is repeated if at least S of the $S \times T$ trials led to an accepted move, i.e. the partition was changed at least S times. If less than S moves were made in the $S \times T$ trials, the value of S is reduced by 10 and the value of T is multiplied by 10, and new rounds of trials are started. This is continued until S falls below 10, after which the MCMC moves are stopped. Note that the algorithm keeps track of the partition with the highest likelihood it has seen at any point during the sampling, and sets the partition to this highest likelihood partition at the end of the rounds of MCMC moves. By default we set $S = C$, i.e. equal to the number of cells C, and $T = 1000$, but these values can be changed by the user.

After the MCMC sampling has completed, a final uphill walk is performed as follows. For each pair of clusters existing in the partition, we calculate the likelihood change that would occur if the clusters were merged into one. We then iteratively merge clusters until no more mergers are left that would increase the likelihood. Finally, for each cell we calculate the likelihood change that would occur if the cell were moved into any of the currently existing clusters, and move the cell to the cluster that maximizes the likelihood.

It is important to note that, although the MCMC sampling scheme is designed to sample partitions in proportion to their likelihood, in practice we find that $P_{\text{move}} * P_{\text{bias}}$ is almost always either very large, or very small. Therefore, in practice this sampling scheme effectively performs a random uphill walk.

B2 Simulated Datasets

The simulated datasets were created based on the inferred cellstates of real datasets. Of the 36 datasets which were analyzed for this paper, we selected those 18 that have less than 6000 cells, more than 3 cellstates with more than 10 cells in them, and a median number of UMIs per cell greater than 1000. Our aim was to simulate datasets based on the set of transcription quotients inferred to be present in the real datasets. Additionally, we wanted to make sure that the clusters can only be identified by differences in transcription quotients (i.e. relative gene expression levels) and not by differences in total UMI counts. The total number of UMI for each cell c, N_c, was therefore drawn independently from a log-normal distribution that was fitted to the experimental distribution of N_c in the corresponding dataset.

For each cellstate s, we determined the mean expression transcription quotient vector $\langle \vec{\alpha}_s \rangle$ and then sampled the UMI count vectors $\vec{n_c}$ of each cell c in the cellstate s from a multinomial distribution with mean $\langle \vec{\alpha}_s \rangle$. However, we found that the maximal likelihood partition of the simulated dataset often differed from the partition generating the dataset, especially for very small and singlet clusters. In particular, when cells that were in a singlet state were given a lower total UMI count in the simulation, these cells were often no longer statistically significantly different from other states, and this to a lesser extent also affected small clusters. To mitigate this problem, we retained only cellstates with more than 10 cells. The number of cells in each cellstate was kept the same as in the experimental data. With this simulation procedure, we made sure that most simulated cellstates would be statistically distinct, even when total UMI counts were lower than in the original dataset.

In this way, three separate simulated datasets were generated for each experimental dataset. Lastly, an additional three "down-sampled" simulations were carried out with $\langle \log_{10}(N_c) \rangle = 3$ and $\text{var}(\log_{10}(N_c)) = 0.1$ fixed for all datasets.

B3 Further Discussion of the results on simulated data

In Figure B we show, for each of the three simulations (marked by their color) generated per experimental dataset (different columns), the detailed outcomes of three independent Cellstates runs per simulation. The top panel shows the difference in log-likelihood ΔL between the inferred partition and the partition used to generate the simulated data. Negative scores mean that the inferred partition had a lower likelihood than the simulated one and can be attributed to a failure of the algorithm to find the optimal partition.

As can be seen, such errors are rare and there is always at least one out of the three runs that has $\Delta L \geq 0$, i.e. a partition at least as good as the one used to generate the dataset was always found. Positive scores indicate that a partition was found with a higher likelihood than the one used to generate the dataset. This can happen for example if there are not enough counts in the simulated cells to statistically distinguish cellstates. To test this hypothesis, we looked at the "down-sampled" simulations with fewer UMI per cell which would make cells with similar, but distinct transcription quotients indistinguishable. Indeed, the results shown in Figure C confirm this hypothesis: For most down-sampled simulations, the best-scoring partition is different from the ground-truth. Also, they tend to score low on homogeneity but high on completeness - which means that inferred clusters are unions of clusters used to generate the simulated data.

C Benchmarking of other clustering tools

For each dataset we started from the raw UMI count table. Cellstates was run directly on the UMI count table and obtained the optimal partition ρ_*. In addition, we hierarchically merged cellstate clusters until the number of clusters matched the number of clusters in the reference annotation for the corresponding dataset.

For all other clustering tools we first normalized and log-transfered the data as follows. Let n_{gc} be the raw UMI count for gene g in cell c, $N_c = \sum_g N_{gc}$ the total UMI count of cell c, and N_{med} the median of N_c across the cells in the dataset. We then obtain the normalized expression levels

$$x_{gc} = \log \left[N_{\text{med}} \frac{n_{gc}}{N_c} + 1 \right]. \tag{46}$$

Each of the clustering tools was then run on the matrix of expression levels x_{gc} using all default parameters. However, for most algorithms the user needs to provide a parameter to control the number of clusters. We set this parameter for each method so as to set the number of clusters to the number of clusters in the annotation of the corresponding dataset.

Finally, since SuperCell is designed to produce fine-grained clusterings, we obtained two separate partitions for this method for each dataset: one with the number of clusters matching the number of clusters that Cellstates produced on the dataset and one where the number of clusters matched the number of clusters in the reference annotation.

The performance was assessed by calculating homogeneity and completeness for the partitions of each method on each dataset.

D Substructure within cell types found by Cellstates does not correspond to batch effects

It is conceivable that the detailed structure in gene expression states found by Cellstates could reflect batch effects to some extent, e.g. cells from different batches could consistently separate into different cellstates. To investigate to what extent cells from different batches separate into different cellstates we used two datasets. First, the dataset of Zeisel et al. [4] contains cells from batches of animals with different age and sex and we investigated the composition of each cellstate in terms of age (younger or older than 24 months) or sex (male, female, or not determined). We find that cellstates that contain multiple cells generally correspond to mixtures cells of different age and sex. In particular, Fig. K shows the sex and age composition of all interneuron cellstates.

To confirm that this observation extends to other datasets we used a dataset consisting of eight samples from the ventricular-subventricular zone (V-SVZ) of the mouse brain that were single-cell sequenced using DropSeq [5]. Of the eight samples four were from females and four from males. Furthermore, two replicate

samples where from the lateral wall (LW) and two from the septal wall (SW) of the V-SVZ from each sex. We ran Cellstates with default parameters on the raw matrix of UMI counts across genes and cells and constructed the hierarchical tree of cellstates. Figure L shows the resulting hierarchy of cellstates together with the composition of the clusters in terms of the 8 batches (pie charts in Fig. L). Note that the pie charts are normalized so as to reflect the differing total number of cells from each batch.

One can observe that, also for this dataset, most cellstates are relatively balanced in terms of the batches from which the cells derive. For example pericytes and venous endothelial cells show a roughly uniform distribution over all samples. In contrast neuroblasts as well as microglia show a more biased distribution. In the case of neuroblasts we can see an enrichment in the LW samples, which is expected since the LW is more neurogenic than the SW. One can furthermore observe a few highly biased clusters, sometimes being almost entirely comprised from one sample. However, we did not manage to unambiguously assign a known cell type to these clusters.

In summary, these analysis support that cellstates do not reflect batch effects and that cells from different batches commonly mix within single cellstates.

E Supplementary Figures

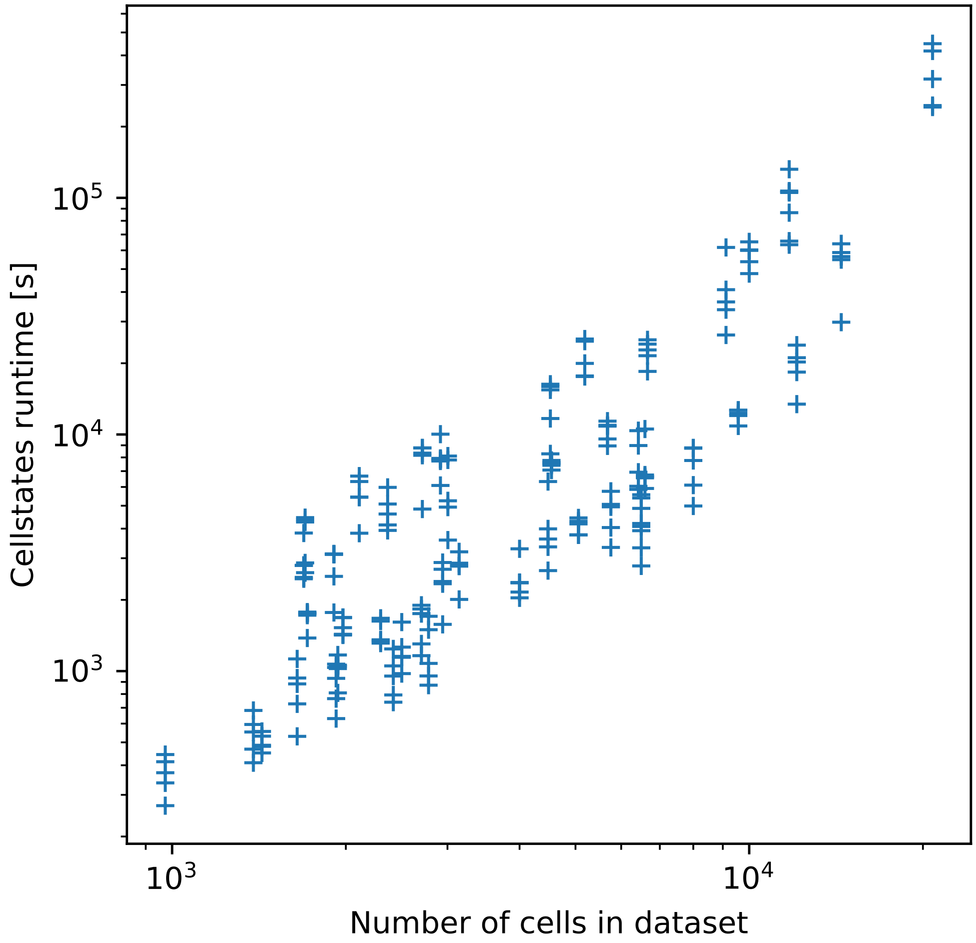

Fig A. Runtimes of Cellstates (vertical axis) as a function of the number of cells in the dataset (horizontal axis) for all datasets analyzed in this study. Note that both axes are shown on a logarithmic scale.

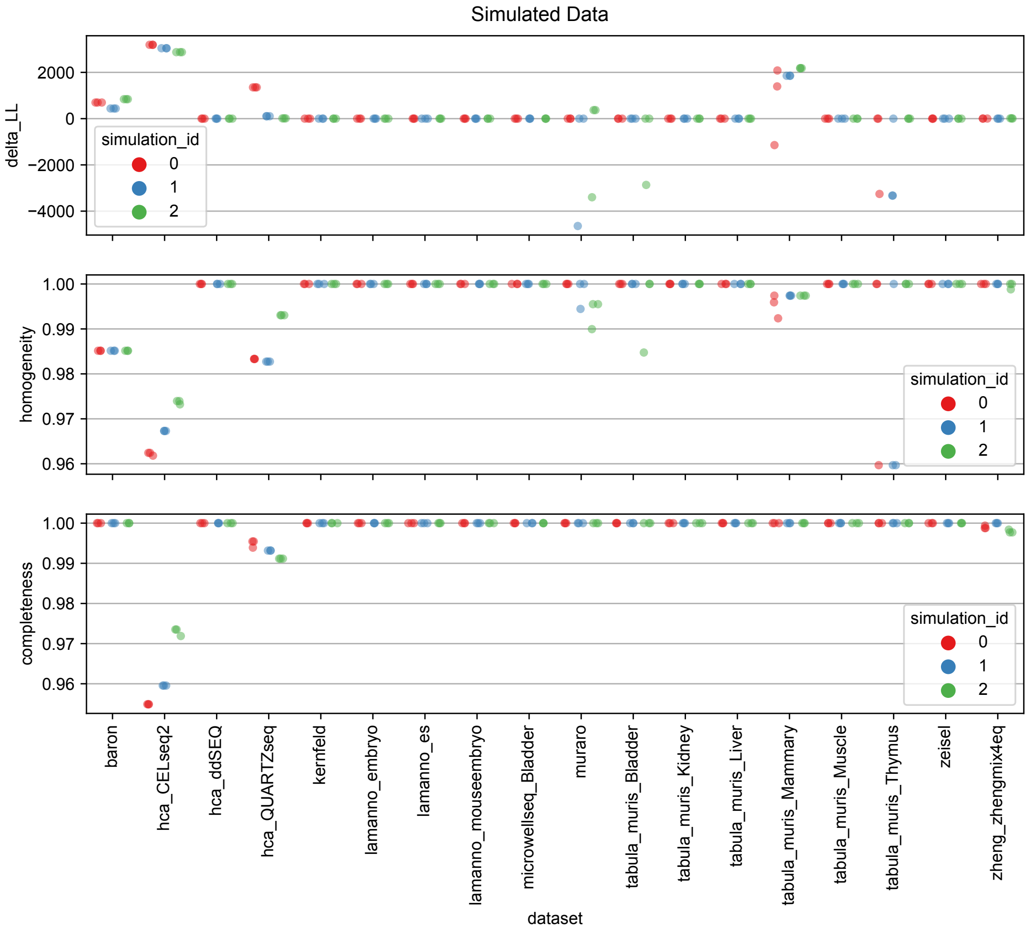

Fig B. Detailed results from CELLSTATES runs on simulated data based on the set of GESs from various indicated datasets. For each of the 18 real datasets, three simulations were generated (red, blue, and green) and CELLSTATES was run three times on each simulated dataset. In the top panel, the difference in log-likelihood delta_LL between the inferred and simulated partitions is shown (with a positive difference meaning that a partition was found with higher log-likelihood than the one used to simulate the data). The corresponding homogeneity and completeness of the inferred compared to the simulated partitions are given in the two lower panels.

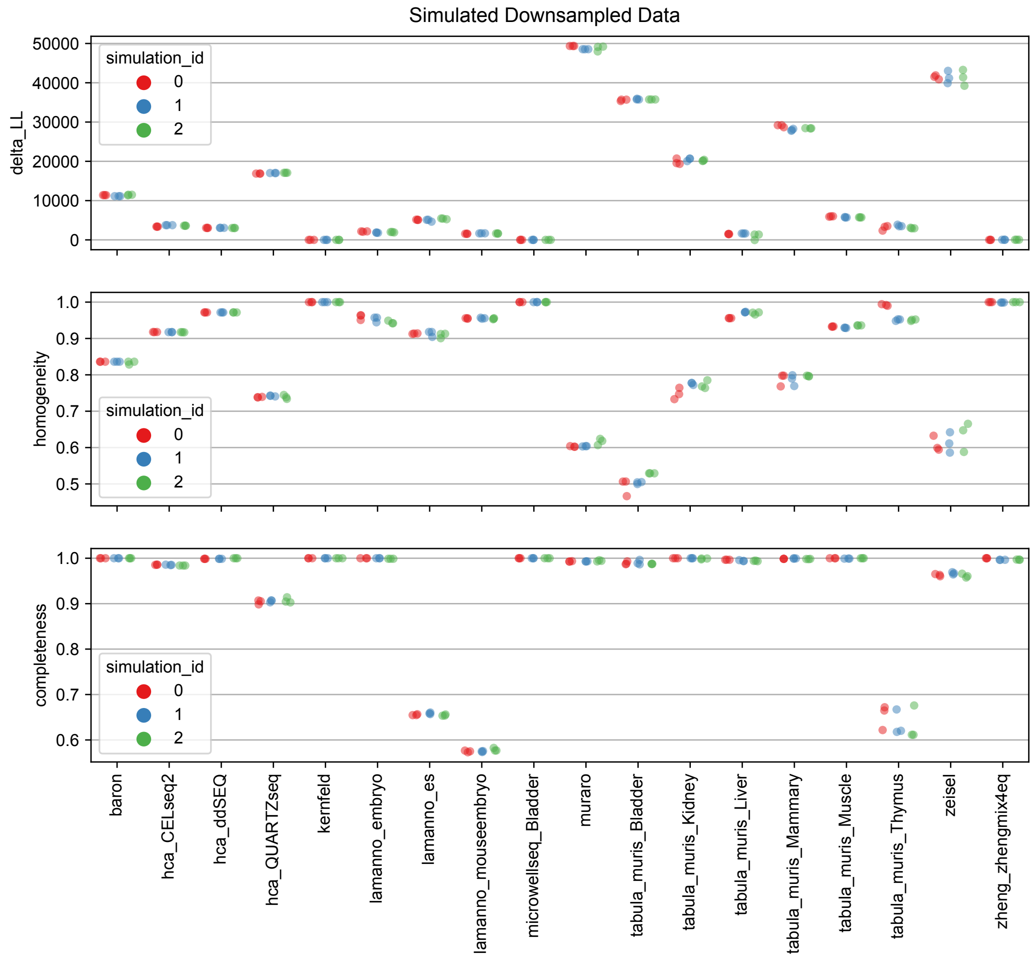

Fig C. Detailed results from CELLSTATES runs on down-sampled simulated data based on the set of GESs from various indicated datasets, but with a median of only 1000 UMI per cell. For each of the 18 GES-sets, three simulations were generated (red, blue, and green) and CELLSTATES was run three times on each simulation. In the top panel, the difference in log-likelihood delta_LL between the inferred partition and the partition used to generate the simulated data is shown (positive meaning that a partition was found with higher log-likelihood than the one used to generate the data). The corresponding homogeneity and completeness of the inferred partitions compared to the partitions used to generate the data are shown in the two lower two panels.

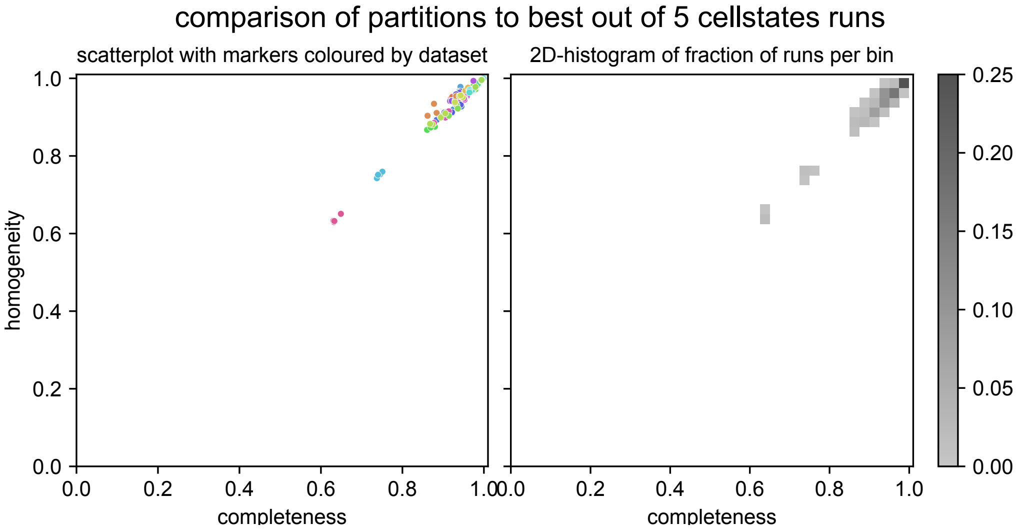

Fig D. Reproducibility of independent Cellstates runs. Cellstates was run 5 times on each of 34 scRNA-seq datasets and the highest-likelihood partition that was found was compared with those of the 4 other runs. The resulting homogeneity and completeness scores are shown twice. On the left as a scatter-plot with markers colored by dataset, illustrating which outliers belong to the same dataset. On the right, a 2D-histogram shows the fraction of runs in bins of size 0.025×0.025, showing that most fall within the top right corner with homogeneity and completeness > 0.95.

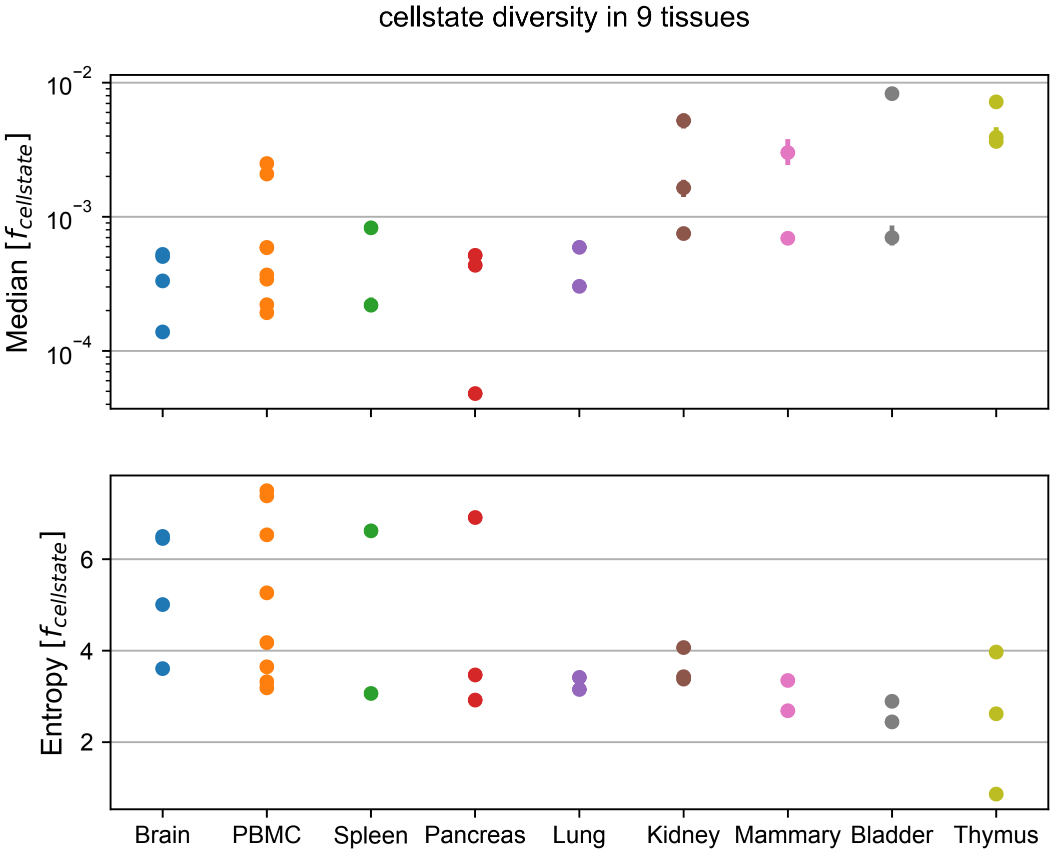

Fig E. Tissue of origin is predictive for cellstate diversity. For 29 different datasets from 9 tissues the median cellstate abundance $f_{\mathrm{cellstate}}$ (top panel) and the entropy of the distribution of cellstate abundances is shown stratified by tissue (horizontal axis). Although there is large variability across datasets, datasets from the same tissue tend to have similar cellstate diversity. Tissues are sorted roughly from most to least diverse from left to right.

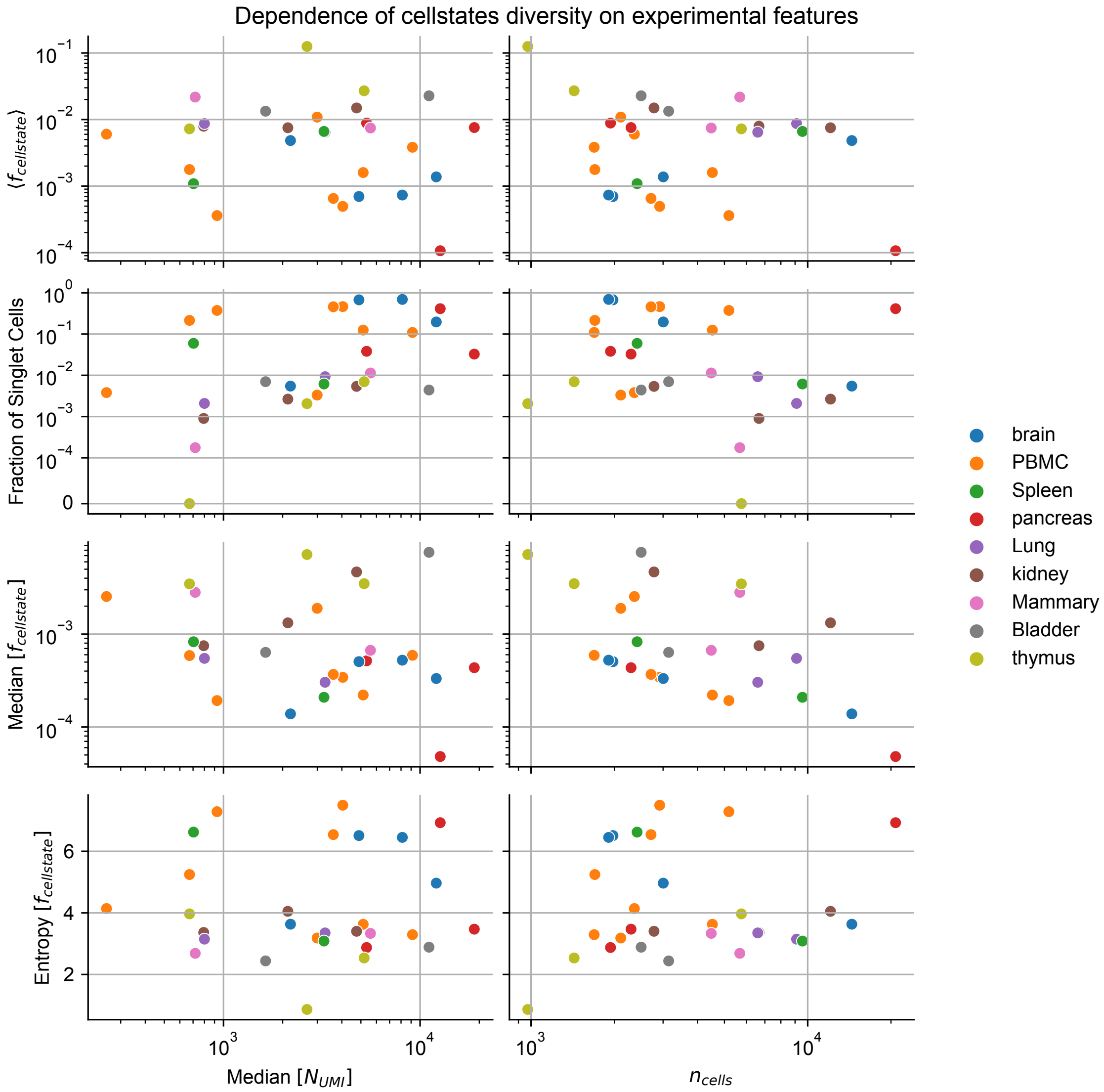

Fig F. Cellstate diversity does not depend on technical features of the experiment. The mean of the distribution of cellstate abundances $f_{\text{cellstate}}$ (top row panels), the fraction of singlet cellstates (second row panels), the median (third row panels) and the entropy (bottom row panels) of $f_{\text{cellstate}}$ are shown as a function of the median total number of UMIs per cell (N_{UMI}) (left column panels) and the total number of cells in each dataset (right column panels). Marker colors indicate the tissue from which the samples originate. These results show that there is no correlation between the various diversity measures and either sequencing depth (total UMI count) or the number of cells sequenced, with the exception of a weak negative correlation between the median cellstate abundance Median[$f_{\text{cellstates}}$] and the number of cells n_{cells}. This correlation is explained by the fact that the majority of cellstates are singlets in many experiments.

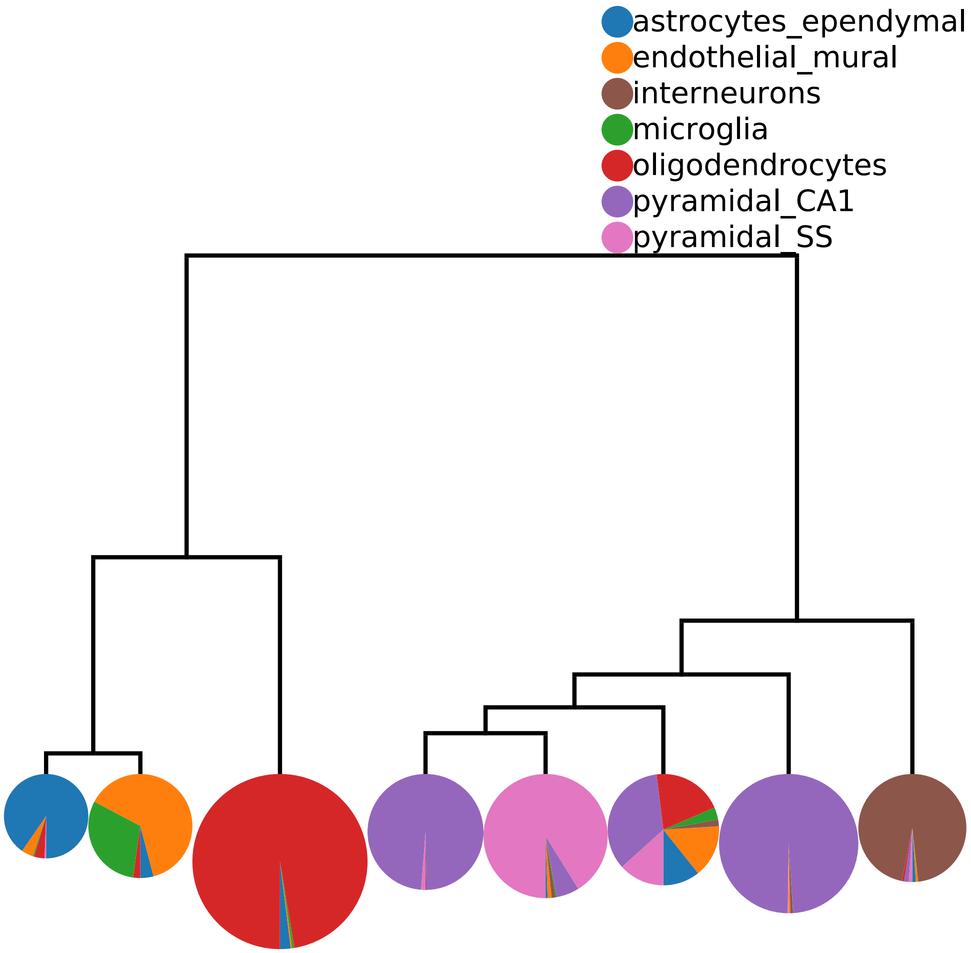

Fig G. Cells in the 8 major cellstate clusters of the Zeisel dataset [4] largely match annotations provided in that publication. Colors indicate different annotations from [4] (see legend), each pie chart corresponds to one higher-order cluster, and the area in each pie chart is proportional to the number of cells with the corresponding annotation in that cluster. Note that cells annotated by [4] as microglia and endothelial-mural cells are merged into one cluster at this level of the hierarchy. The tree structure indicates how these clusters are related upon further merging.

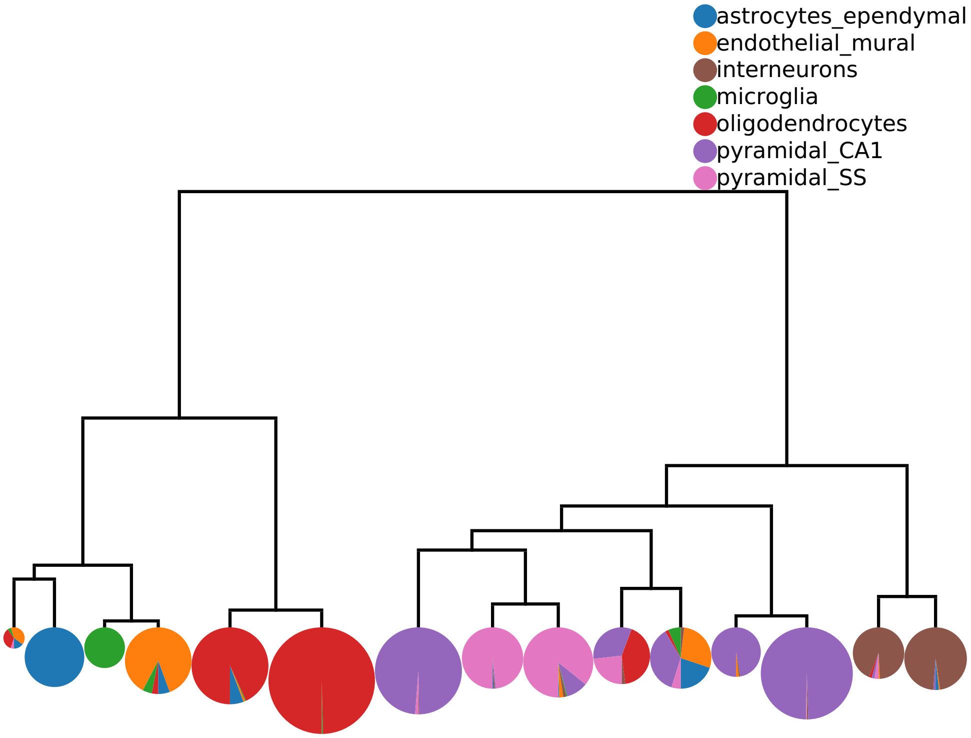

Fig H. At 15 higher-order cellstate clusters cells annotated in [4] as microglia and endothelial-mural separate. Colors indicate different annotations from [4] (see legend) and the area in each pie chart is proportional to the number of cells in the corresponding cluster with the corresponding annotation. Note that at this level of the hierarchy cells with a common annotation in [4] tend to separate into multiple higher-order cellstate clusters.

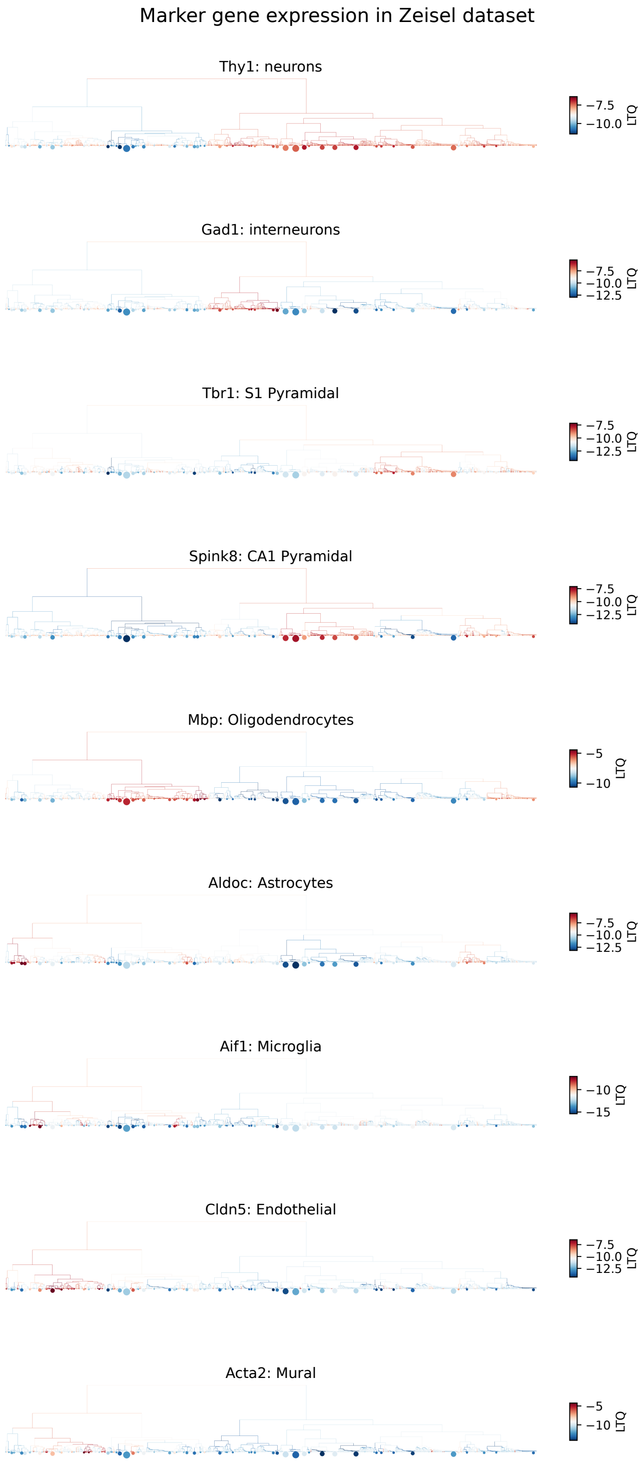

Fig I. Expression of marker genes for annotated cell types visualized in the hierarchical tree of higher-order cellstate clusters. The sizes of the discs at the leaves correspond to the numbers of cells in the corresponding cellstates. See Figure 4 to compare with the higher-order clusters of Cellstates.

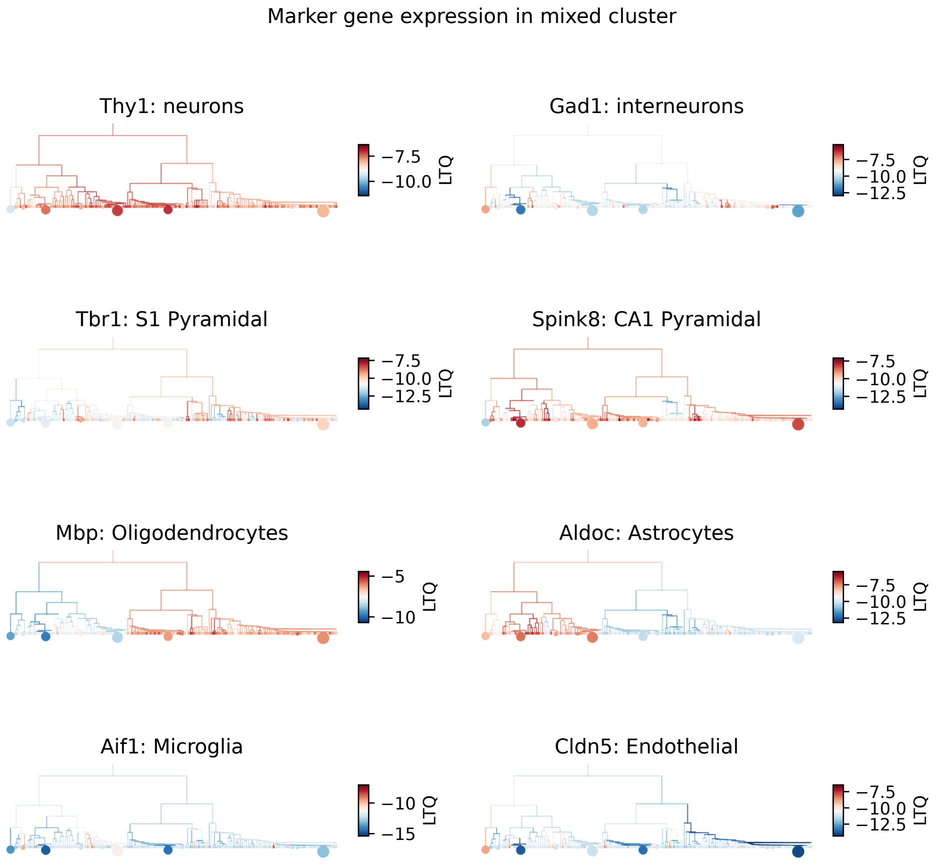

Fig J. Expression of marker genes for annotated cell types visualized in the hierarchical tree of higher-order cellstate clusters. The sizes of the discs at the leaves correspond to the numbers of cells in the corresponding cellstates. See Figure 4 to compare with the higher-order clusters of Cellstates.

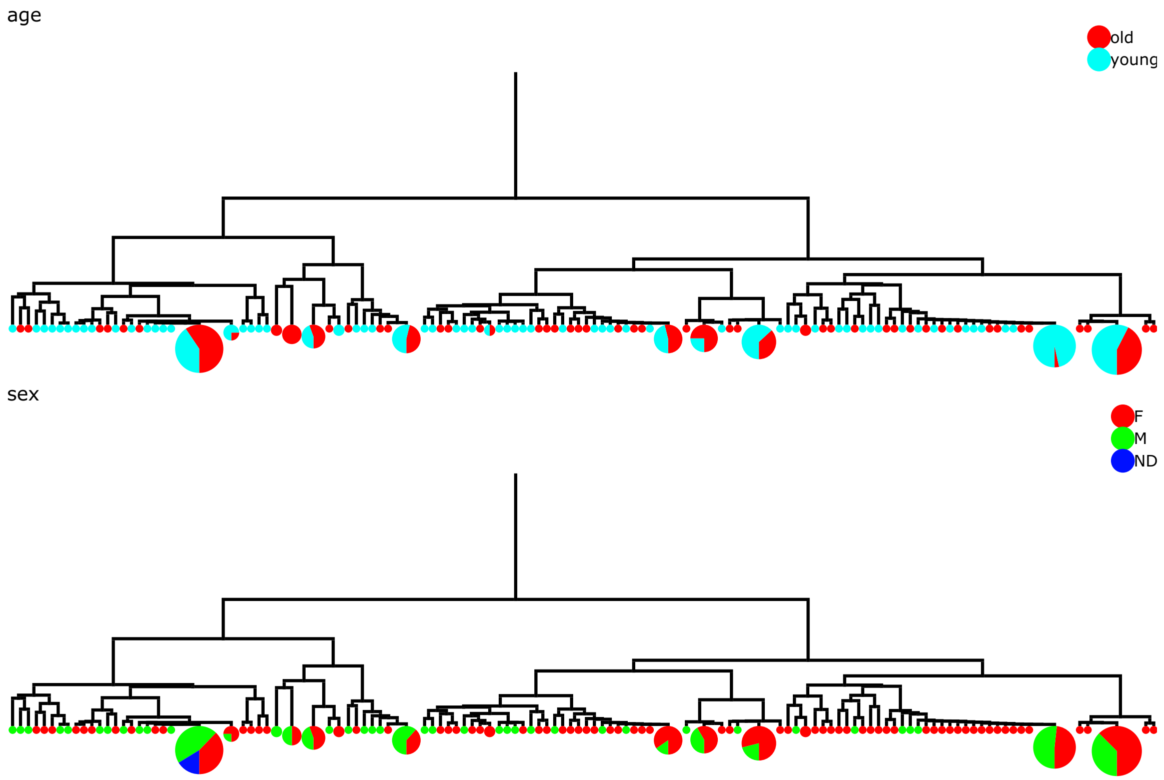

Fig K. Cells in cellstates of the Zeisel dataset [4] derive from batches with different age and sex. Shown is the sample composition in terms of age of the donor (i.e. either older or younger than 24 months of age, top panel) or its sex (bottom panel) for all the interneuron cellstates. The sizes of the discs at the leaves correspond to the numbers of cells in the corresponding cellstates and the colors indicate the fractions of cells of coming from old/young or male/female donors. Note that almost all cellstates that contain more than one cell consist of mixtures from different batches.

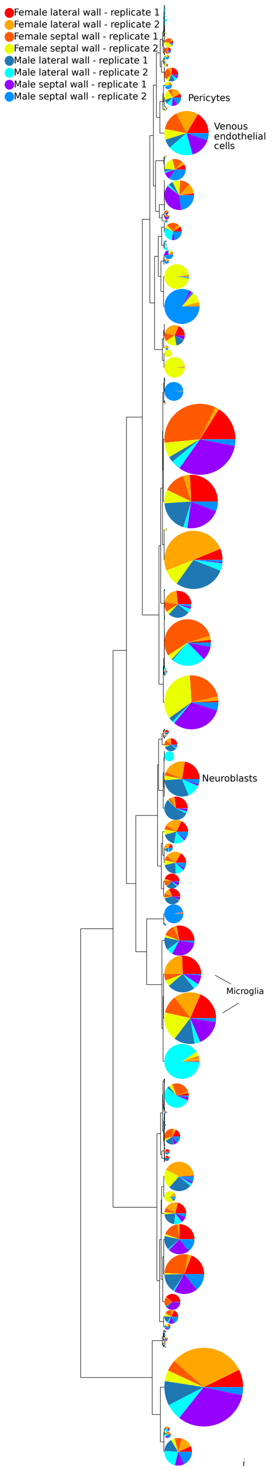

Fig L. Hierarchical tree of cellstates for the data of [5] consisting of cells of 8 samples from the ventricular-subventricular zone (V-SVZ). The pie chart of each cellstate indicates the composition of its cells from the 8 different batches, which derived from individuals of different sex and corresponding to different locations in the V-SVZ (see legend). The figure shows that most cellstates correspond to relatively well balanced mixtures of cells from the different samples. Note that the pie charts are normalized to reflect the total number of cells from different batches in the data.

References

1. Breda J, Zavolan M, van Nimwegen E. Bayesian inference of gene expression states from single-cell RNA-seq data. Nature Biotechnology. 2021;doi:10.1038/s41587-021-00875-x.

2. Phillips R, Kondev J, Theriot J, Garcia HG, Orme N. Physical Biology of the Cell. Boca Raton: Garland Science; 2012.

3. van Nimwegen E, Zavolan M, Rajewsky N, Siggia ED. Probabilistic clustering of sequences: Inferring new bacterial regulons by comparative genomics. Proceedings of the National Academy of Sciences. 2002;99(11):7323–7328. doi:10.1073/pnas.112690399.

4. Zeisel A, Muñoz-Manchado AB, Codeluppi S, Lönnerberg P, Manno GL, Juréus A, et al. Cell types in the mouse cortex and hippocampus revealed by single-cell RNA-seq. Science. 2015;347(6226):1138–1142. doi:10.1126/science.aaa1934.

5. Mizrak D, Levitin HM, Delgado AC, Crotet V, Yuan J, Chaker Z, et al. Single-Cell Analysis of Regional Differences in Adult V-SVZ Neural Stem Cell Lineages. Cell Rep. 2019;26(2):394–406.
