# Supplementary material for: Identifying cell states in single-cell RNA-seq data at statistically maximal resolution
Source: PLoS Comput Biol. 2024 Jul 12;20(7):e1012224. doi: 10.1371/journal.pcbi.1012224 (PMC11364423; doi:10.1371/journal.pcbi.1012224)
Supplement: Attachment — Submitted filename: response_reviews.pdf [file pcbi.1012224.s004.pdf]

Reponse to reviewer comments

Basel, April 30, 2024

Dear editor,

We are hereby submitting a revised manuscript of our manuscript entitled 'Identifying cell states in single-cell RNA-seq data at statistically maximal resolution'. We were very pleased with the highly constructive comments of the reviewers and have followed most of their suggestions in our revisions, including a number of additional analyses along the lines suggested by the reviewers. Below we are providing detailed point-by-point responses to the reviewer comments and here just briefly summarize our revisions:

1. Following a suggestion of reviewer 1, we have now added comparison with the SuperCell method to the benchmarking of Figure 2.

2. Following suggestions from both reviewers, we have now added analyses of batch effects. In particular, for two datasets that consist of cells from multiple batches, i.e. donors and samples from different parts of a given tissue, we have analyzed the composition of the Cellstates in terms of these batches (Suppl. Figs S11 and S12 and Suppl. Information section A.4). This analysis shows that most cellstates consist of fairly balanced mixtures of the different batches, supporting that different cellstates do not generally reflect different batches.

3. Following a suggestion of reviewer 1, we have added a new method for finding marker genes (described in Suppl. Information section A.1.5). Example applications of this method have been added to the Github pages of Cellstates.

4. Following a suggestion of reviewer 2, we have added a figure with runtimes of Cellstates on the datasets analyzed in this manuscript (Suppl Fig S1).

5. To facilitate analysis of very large datasets, we have added a method that allows running Cellstates incrementally on portions of large datasets, and allows restarting of Cellstates from intermediate results. Example use of these methods has been added to the Github pages as well.

6. We have added color scales to Suppl. Figs. S9 and S10.

7. Using feedback from the reviewers, we have rewritten parts of the manuscript that the reviewers found not sufficiently clear, and have added citations.

Note that several of the additional analyses presented in the revision were performed by Thomas Sakoparnig, who has been added as an author.

Together we feel that these revisions and additional analyses have significantly strengthened the paper and we thank the reviewers for their suggestions. We believe that we have addressed all the concerns of the reviewers and hope that you will now find our manuscript acceptable for publication.

Thank you for your consideration and best regards,

Erik van Nimwegen

Detailed responses to the comments of reviewer #1

Comment 1.1: *Even though methodologically different, the aim of the method is highly analogous to the MetaCell approach (Baran et al. Genome Res 2019; https://doi.org/10.1186/s13059-019-1812-2) that states in its abstract: "We describe a methodology for partitioning scRNA-seq datasets into metacells: disjoint and homogenous groups of profiles that could have been resampled from the same cell.". Due to this overlap in the methods' objectives, authors should add MetaCell to the set of benchmark methods (Fig 2). What is the advantage of Cellstates over MetaCell?*

Response 1.1: In selected clustering methods to compare Cellstates with, we focused on methods that had clear default settings, and avoided methods that required a large number of parameters to be set manually. Unfortunately, the MetaCell method falls in this latter category. However, we understand that reviewer's wish to compare with a method that also aims to only cluster cells with very similar expresison profiles. A recently published method, called SuperCell, that takes an approach very similar to MetaCell, and was reported to outperform MetaCell. This method also has clear default settings (apart form a granularity level that needs to be set by the user).

For the revision we have now included a comparison with SuperCell in Figure 2B. This analysis show that, Cellstates also clearly outperforms SuperCell. In particular, Cellstates has the highest homogeneity of all methods in all datasets, and although SuperCell often reaches an homogeneity that is almost as high as Cellstates', it always has substantially lower completeness.

Comment 1.2: *One major drawback for biologically meaningful usage of the method is the lack of addressing batch effects. The authors should show usage of the method on data compiled of several distinct batches (from distinct studies) of the same tissue to test whether there are any "cellstates" shared across the batches. If not, the term "cellstate" is misleading and should be replaced; for example the MetaCell concept discussed above is more realistic in this sense. 'Cell state' is a useful concept only if there are shared states across samples and tissues from distinct individuals; otherwise it would not be possible to make any generalizations of the functional potential of each state.*

Response 1.2: As also discussed in a recent work on scRNA-seq analysis from our lab (Breda et al Nat Biotech 2022), the term 'batch effect' is used in the literature to refer to a wide array of uncontrolled variations in experiments of both technical and biological nature. In our opinion, analysis methods should not conflate true biological differences in gene expression (for example due to different genotypes or experimental conditions) from differences that result from technical variation in the experimental protocols. Thus, in our opinion the task that Cellstates is solving, i.e. to divide the cells into groups with statistically indistinguishable gene expression states, is really independent of whether the observed differences in gene expression states are entirely biological in nature, or whether they to some extent derive from technical variations.

However, we do agree with the reviewer that it would be problematic if it were the case that the cells within cellstates are always exclusively coming from individual batches. Fortunately, we find that this is clearly *not* the case and in the revision we have added supplementary analysis showing that, for two example datasets where we have batch annotations, most cellstates consist of mixtures of cells from different batches.

In particular, for the Zeisel dataset, different batches derive from individuals with different ages (which we classified as either older or younger than 24 months) and different sex. In

Supplementary Figure S11 we now show the composition of all interneuron cellstates by age and sex of the donors, which confirms that cellstates typically consist of mixtures of cells from different donors.

As a second example, we analyzed a dataset of Mizrak *et al.* consisting of single cells from the ventricular-subventricular zone (V-SVZ) of mouse cerebellum that came from 8 different samples, with 4 from males and 4 from females, and from different locations of the V-SVZ (4 from the lateral wall and 4 from the septal wall). We show that the cellstates inferred on this dataset also generally show fairly balanced mixtures from these different batches (Suppl Fig. S12).

These new results show that, for datasets combining different batches, cellstates consist of mixtures of cells from different batches.

Comment 1.3: *In the Discussion section, the authors suggest that as the "cellstate" number does not increase proportionally as the number of cells increases, cell states are in fact discrete rather than continuous. They also state that especially in more complex tissues, a large number of "cellstates" are formed by individual cells, suggesting that cell states are not sampled until saturation, as they also find larger cell clusters of statistically identical cells. However, the observation that the number of "cellstates" depends more on tissue (complexity) than on number of cells does not suggest against the continuum model. It could also be that the continuum is shorter for the less complex tissues, and that the continuums have more and less densely populated regions (some of which could be even extinct by the time of sampling). In this paradigm, the dense regions (analogous to valleys in Waddington landscapes) composed of larger number of statistically identical cells would be identified as "cellstates" as they are more likely to be sampled than sparser regions. Please improve the Discussion section on this matter.*

Response 1.3: We understand the confusion. We agree that, conceptually, one can think of the gene expression states of cells as deriving from a continuous density per definition, and think of the 'discrete' cellstates that our algorithm finds as local maxima in this density that are so 'tight' that different states around these local maxima are statistically indistinguishable (even if all mRNAs were captured). When we used the term 'continuum' in the Discussion section, we were thinking of a continuous distribution of states that is extensive enough so that many statistically distinguishable states occur in it.

For the revision we have rewritten and expanded this part of the discussion to avoid misunderstanding.

Comment 1.4: *"It should be noted that the most significant genes are those for which the average expression on opposite sides of the branch is most significantly different, but the expression of these genes might be quite variable across the cellstates below each branch."*

This pseudobulk type of approach of comparing averages can lead to rather spurious results, especially when considering that each "cellstate" can be composed of only a small number of cells. Please either justify this choice or improve the approach to extract markers?

Response 1.4: We agree that this is a valid concern and we did not properly explain the purpose of this significance score. The higher-order clusters are generated using the same likelihood function as is used by Cellstates to infer the optimal partition, i.e. cellstates are merged so as to minimize the decrease in the posterior probability of the partition and these significance scores are simply quantifying how much each gene contributes to this decrease in log-likelihood when two groups of cellstates are merged. The purpose is thus to, within one

rigorous probabilistic framework, identify which genes most significantly distinguish cell states at each split in the hierarchy.

However, we completely agree with the reviewer that if one wants to find genes whose expression 'marks' a particular subset of cells, then this scoring function is not satisfactory since it essentially only measures the statistical significance of the difference in *average* expression of the gene on opposite sides of the split. But a significant difference in average does not guarantee that the distributions of expression levels on opposite sides of the split are non-overlapping.

Finding genes that 'mark' subsets of cells in the sense that the distribution of expression levels in the subset does not overlap with the distribution of expression in other cells is a conceptually different problem but we agree this is also an important problem. In fact, after submission of our manuscript we have developed a probabilistic method to identify genes whose expression best distinguishes between two sets of cellstates and implemented it in C code that we have already been using ourselves.

For the revision we added a section in the supplement describing this new method for identifying marker genes (section A.1.5). We have made our implementation of this method available via the Cellstates pages on Github, and also provide notebooks with examples of its use. Finally, we have edited the manuscript to better explain the difference between our method for finding significantly differentially expressed genes and our new method for identifying marker genes.

We thank the reviewer for bringing up this issue because we believe this added functionality has significantly strenghtened Cellstates.

Comment 1.5:*Please add citations to the referred 18 experimental datasets used as basis for the simulation to the Results where these data are first mentioned.*

Response 1.5: Thank you. This is a good suggestion and we have now included these citations in the revision.

Detailed responses to the comments of reviewer #2

Comment 2.1: *The authors should qualify their statements about batch effects in Section 3.4 and in the Discussion further. While they report that cellstate diversity reflects inter-tissue variability rather than technical variability in Section 3.4, they should expand their analysis to consider inter-donor variation within each of their datasets.*

Response 2.1: As we also mentioned in response 1.2 to a comment from reviewer 1, the term 'batch effect' is used rather loosely in the literature to refer to uncontrolled variations that may result from both technical and real biological variability and it is not obvious which of these effects should be 'corrected' for in the scRNA-seq analysis. For example, it is certainly possible that cells of a given type from donor A are systematically different in gene expression from cells of the same type of donor B (for example due to genetic differences between donors A and B). Such true biological variation might in fact contain important information that an analysis method should arguably not normalize away.

But we agree with the reviewer that, in addition to the analysis that was presented in section 3.4, it is informative to investigate how cellstates vary across donors in experimental datasets deriving from multiple donors. For the revision we have now performed such analysis for two example datasets, i.e. as already mentioned in response 1.2:

In particular, for the Zeisel dataset, different batches derive from individuals with different

ages (which we classified as either older or younger than 24 months) and different sex. In Supplementary Figure S11 we now show the composition of interneuron cellstates by age and sex of the donors, which confirms that cellstates typically consist of mixtures of cells from different donors.

As a second example, we analyzed a dataset of Mizrak et al consisting of single cells from the ventricular-subventricular zone (V-SVZ) of mouse cerebellum that came from 8 different samples, with 4 from males and 4 from females, and from different locations of the V-SVZ (4 from the lateral wall and 4 from the septal wall). We show that the cellstates inferred on this dataset also generally show fairly balanced mixtures from these different batches (Suppl Fig. S12).

These analyses show that, at least for these datasets, cells from different donors are sufficiently similar in their gene expression patterns such that individual cellstates typically consist of mixtures of cells from different donors.

Comment 2.2: *The authors should validate Cellstates on a wider range of datasets. The paper primarily focuses on the application of the Cellstates tool on specific datasets. Expanding this analysis to a wider variety of datasets, especially those with varying complexities, could enhance the generalizability and robustness of the conclusions. Specifically, there are several common scenarios in scRNA-seq data analysis where Cellstates could identify subtle substructure within cell types, which would exemplify its relevance to a broader readership. One such setting is in cancer datasets, where malignant cells are often aneuploid and can result in correlated changes in expression of neighboring genes that are simultaneously affected by copy number aberrations. These typically result in donor-specific gene expression states, and thus Cellstates may be well positioned to directly identify such states. There is a vast literature reporting scRNA-seq datasets in genomically unstable cancers where they could find some test datasets to try this (e.g. PMID: 36517593, PMID: 29674595). Another such setting is in the study of immune dysfunction, which has been explored in the context of aging, infection and cancer. Specifically, the progression of T cells across a phenotypic continuum traversing discrete cell states (naive, effector/memory, pre-dysfunctional, dysfunctional) is hotly debated in the field. There is also substantial literature on this topic where they can find example datasets (e.g. PMID: 30595452).*

Response 2.2: We agree with the reviewer that there are many potential applications of Cellstates that would be interesting to pursue, including mapping cellstates of genomically unstable cancers, immune dysfunction, and aging. However, such applications are well beyond the scope of this manuscript. Note that although it is very straight forward to run Cellstates on datasets from cancers or immune cells traversing different states, presenting a thorough analysis of the results and giving biological interpretations of what we find would constitute an entire separate study. Including this would not only be an enormous amount work, it would fundamentally change the nature of our study.

The topic of our paper is to introduce the Cellstates method, explain its motivation, and explain how the algorithm is derived from first principles. We further demonstate the power and validity of the method using a wide selection of simulated and real datasets. We note that this also includes datasets with large variation in their complexity, as illustrated in Figure 3, which shows that the average cellstate abundances across 29 datasets from 9 tissues vary over more than 3 orders of magnitude, and that the fraction of singlets varies over almost 4 orders of magniture. In addition the median UMI count varies almost 100-fold across these datasets, and the number of cells about 20-fold (Supplementary Figure S5).

Comment 2.3: *The authors advocate for slow and rigorous data analysis, which we fully agree with. Given the increase in our ability to profile hundreds of thousands to millions of cells by scRNA-seq nowadays however, they should include results of the runtime on their benchmarking datasets with variable number of cells. It would be great if they could also include some recommendations for users aiming to apply their tool to large datasets, who may for example resort to analyzing subsets of a dataset with Cellstates which may not guarantee the same partitions.*

Response 2.3: We agree with the reviewer. In the revision we now provide runtimes of cellstates as a function of the number of cells on the benchmarking datasets (Suppl. Fig. S1). This shows that runtimes can vary 10-fold for different datasets with the same number of cells.

In addition, to help users that wish to run Cellstates on very large datasets, we implemented a method that allows running Cellstates incrementally. In particular, this method allows users to add a set of cells to a given partition of another set of cells, and obtain a joined optimal partition. In this way, users can divide a large dataset into subsets of cells that can be partitioned incrementally. Although such incremental clustering can lead to less optimal results, it does allow running of very large datasets that would otherwise be impractical. This new method is described in the revision and provided via the Github pages of Cellstates, where we have also provided notebooks with example application.

Comment 2.4: *Figures S8 and S9 are missing a color scale.*

Response 2.4: We thank the reviewer for noting this omission. In the revision we have added color scales to these figures (now S9 and S10).

Comment 2.5: *It would be helpful to add a table in Supplementary A.1. where all random variables (r.v.s) and their respective symbols are defined. Also, it will help the readability of the methods section to have a table where the probability distribution function of these r.v.s. is noted.*

Response 2.5: A famous theoretical physicist once told one of us that theoreticians would rather use each other's toothbrushes than using each other's mathematical notation. This is a bit how we feel about this request. Reading the reviewer's summary of our work and some of the comments, it is clear to us that this reviewer's preferred style of notation is very different from the one we prefer. For example, the 'random variable' terminology which formally distinguishes beween variables that are unknown (but definite) and parameters that are intrinsically 'random' is foreign to us. Similarly, in our approach 'distributions' reflect a state of information, not frequencies in imagined replicates.

We took quite some care in the presentation of the theory behind Cellstates in the supplementary information and the reviewer does not seem to argue that anything is unclear or ambiguous in this presentation. We thus strongly prefer to stick with our own preferred notation.

Comment 2.6: *It appears that the parameters of the prior are learnt in a data-dependent manner (Θ, the scale factor, and ψ, the simplex.) This is a sensible approach; however, it means that the method falls under the empirical Bayes umbrella. This should be explicitly noted and the relevant papers cited.*

Response 2.6: The term 'empirical Bayes' is used by different researchers to refer to a variety of procedures that differ in their details and we are not sure that there would be broad agreement as to whether the specific procedure used in our paper should or should not be called

'empirical Bayes'. Like the previous request for using random variable and distribution function terminology, we would also like to avoid use of this inprecise terminology.

We believe that we have been very clear and unambiguous in our description of precisely what Cellstates does (i.e. use a Dirichlet family for the priors over the transcription quotient vectors of each cellstate, marginalize over the unknown transcription quotients for each cellstate using this prior, and then jointly maximize over the partition and parameters of this prior).

Comment 2.7: *The writing of the computational section A.2. should be improved. The target posterior should be clearly indicated ($P(\rho, \Theta|D)$?). Then the fact that it is interactable, and therefore MCMC (Metropolis within Gibbs) is used to sample from this posterior.*

Response 2.7: We agree that the presentation in this section was suboptimal and have rewritten this section in the revision.
